# The Institutional Worker

**Published:** 1918-06-01

**Authors:** 


THE
### INSTITUTIONAL WORKER
**Being a Special Supplement to "The Hospital"**

### OUR BUREAU OF INFORMATION.

### Rules for Correspondents.

1. Every letter must be accompanied by the coupon to be cut from the back cover (inside page) of THE HOSPITAL, current issue, and must contain the name and address of the correspondent with pseudonym for publication if desired. Replies by post cannot be given save under exceptional circumstances at the Editor's discretion.

2. Letters from Approved Homes in reply to special needs published in the Bureau should state terms and full particulars, and be sent prepaid under cover to the Editor of the Bureau with name written across coupon for identification.

3. Proprietors of Homes which have not yet been entered on the List of Approved Homes, but have spare accommodation likely to suit special needs, are invited to write for an application form for registration. The fee for registration, which includes two announcements of the Home in the Bureau and other privileges, is 10s.

4. All communications to be addressed to the Editor of THE HOSPITAL, 28 Southampton Street, Strand, London, W.C. 2, and marked "Bureau of Information."

**URGENT NOTICE.**

To ensure a copy of THE HOSPITAL regularly, readers mu t place a definite order with their Newsagent TO-DAY. After the middle of June, in consequence of the new Paper Restrictions Order, it will not be possible for Newsagents to stock copi:s for chance customers.

DO NOT DELAY. ORDER

"THE HOSPITAL" TO-DAY

and obtain your copy regularly EVERY THURSDAY.

### A Register of Nurses' Ward Work.

### Answer to April Question.

**By "Elizabeth."**

**THE question was:**

How should a book be ruled to give at a glance a record of nurses' different duties in male, female and children's wards, and time spent at classes, etc.? The hospital is a training-school, and nurses take day and night duty. There are three floors for male patients, two for female patients, and a children's block.

THE accompanying ruled page shows a method of telling *at a glance* the kind of work a probationer has done, when this information is needed before making a move.

The object is to prove this one point, and no dates are entered beyond the entrance date, the date of completion being added when the full term of training has been covered.

In the first instance nearly two years of training have been covered (the remaining portion of the space would be needed for holidays).

WARD WORK, THREE (OR FOUR) YEARS.

| — | Men's Surgical | Men's Medical | Women's Surgical | Women's Medical | Children's Ward | Special Departments | Classes |
|---|---|---|---|---|---|---|---|
| Margaret Day Jan. 1st, 1900... | 3 months, 1st floor | 2 months | 3 months | 2 months, 2nd floor | 3 months | Theatre, 3 months | Class on General Nursing<br>Surgical Lectures<br>Medical ,,<br>Bandaging Class<br>Cookery ,, |
| | **2 months** | **1 month (extra)** | **1 month (extra)** | — | **2 months** | **Special on Tetanus, 2 weeks** | |
| Catherine Lee Feb. 3rd, 1900... | 2 months, 2nd floor | — | 3 months, 2nd floor | —<br>**2 months** | — | — | Invalid Cookery Class |
| Winifred Clare Feb. 15th, 1900 | — | 2 months, 1st floor. | — | — | 3 months | — | Bandaging Class |
| Ursula Woods March 1st, 1900 | — | — | — | 2 months, 1st floor | — | — | — |

N.B.—Entries in small black type in the lower division denote night duty. Red ink should be used for this purpose.

### JUNE QUESTIONS.

**1. Equipping an X-ray Department.**

Give a list of equipment for an X-Ray, Electrical, and Massage Department in a hospital of 180 beds.

**2. Restrainer for Diphtheria Case.**

Describe a simple, washable restrainer, suitable for helping to keep flat in bed a young child suffering from diphtheria. Such a patient, after the first week or so, does not feel ill, and it is almost impossible to keep a child flat in bed without some kind of restrainer.

### RULES.

The following rules must be observed:—

1. Contributions must be written on one side of the paper. Brevity and terseness are desirable features. The MSS. must bear the name and address of the sender and be accompanied by coupon to be cut from the back cover (inside page) of the current issue of THE HOSPITAL. A pseudonym must be chosen if the name is not to be published.

2. Contributions must be addressed to the Editor of THE HOSPITAL, Institutional Worker Supplement, 28 & 29 Southampton Street, Strand, London, W.C. 2, should reach him before the end of the current month, and be marked in the left-hand corner "Facts and Figures."

**A minimum payment of Five Shillings will be made for each published answer.**

### QUESTIONS INVITED.

In connection with our Question Box, we offer every month five shillings each for the two best questions which are sent in for consideration. The questions may be on any institutional subject and concern any department, from the secretary's to the porter's, or the matron's to the domestic staff; the one essential is that they must be practical and deal with points that have a definite relation to hospital or institutional administration, upkeep, finance, or management. Points relating to artificers' work, housekeeping, the laundry, out-patients, and other practical matters will be welcome. It is hoped that thus, when difficult or doubtful points arise in the course of their work, institutional workers will be encouraged to put them in the form of a question and send them to the Editor, THE HOSPITAL Bureau of Information, 28 & 29 Southampton Street, Strand, W.C. 2, marked "Question Box."

The best questions will be published in due course and our readers will be given the opportunity of answering them. Thus all institutional workers may be able to cooperate with the view of helping the work and smoothing the difficulties of each other. In short, we want the Question Box of the *Institutional Worker* to be freely used by every worker habitually.

### ENQUIRIES AND ANSWERS.

### *WAR MATTERS.*

**The Need for V.A.D. Members.**

WE suggest that you offer your services to the V.A.D. Department of the British Red Cross Society. Address your application to the V.A.D. Department (2), Devonshire House, Piccadilly, W. 1. There is at the present time a serious deficiency of nursing members for naval and military hospitals, and the department is appealing for the services of well-educated and physically fit women between the ages of twenty-one and forty-eight for duty in this country. Previous hospital experience and nursing certificates are not necessary. Those accepted for service receive a salary of £20 a year. —MAY.

**Women Orderlies.**

YES; you can obtain work in a military hospital in France. Women orderlies are urgently required to release male orderlies for the firing line. Applications should be addressed to the V.A.D. Department, Devonshire House, Piccadilly, W. 1.—MORRIS.

**V.A.D.'s Complaint.**

YOU should address your complaint to the commandant of your section, who will forward it to the County Director. Only in the case when the commandant fails to forward the complaint should it be sent direct to headquarters by the V.A.D. member.—TONY.

**A Comfortable Arm Sling.**

A VERY comfortable and serviceable arm sling is made by the Hospital and General Contracts Company, 19-35 Mortimer Street, W. 1., known as the Coutts-Lindsay. It fits the shoulder like a collar and can be readily adjusted to suit any patient, no matter how bulky and cumbersome the dressing may be. The price is 4s. 9d.—COMMANDANT.

**Temporary Post on Hospital Train.**

WE suggest that you address your inquiry as to the possibility of obtaining temporary work on a hospital train to the British Red Cross Society, 83 Pall Mall, S.W. 1.—R. S.

### *THE HOUSEKEEPERS' DEPARTMENT.*

**Recipe for Gingerbread.**

AN excellent recipe for gingerbread is as follows: Mix together 1½ lb. flour, ½ lb. treacle (the dark-brown syrup which is now fairly plentiful will do quite well), 4 oz. of brown cooking sugar or ½ lb. honey, 2 oz. ground ginger, 2 oz. ground allspice, and 1 oz. of carbonate of soda. Mix all these ingredients into an elastic paste and put into a good-sized square tin mould capable of holding it to the thickness of 2 inches. Allow this to stand in a moderate warmth for one hour, and then bake in a moderate oven. This makes a good-sized gingerbread.—HOUSEKEEPER.

### *EMPLOYMENT AND TRAINING.*

**Instruction in Electro-Therapeutics.**

A SPECIAL course in electro-therapeutics, including light treatment, can be taken out at Guy's Hospital, London. Application should be made to the matron. The Zander system of treatment is practised now in many of the military hospitals as well as the Zander Institute, to which you might make application.—W. T. W.

**Training Under the Metropolitan Asylums Board.**

WE think you will find little or no difficulty in obtaining a probationership in one of the infectious hospitals or sanatoria under the Metropolitan Asylums Board. These institutions have been more hardly hit than any in regard to the shortage of nurses for the civil population. The Board's hospitals are divided into three groups: Acute Fever Hospitals, Convalescent Fever Hospitals, River Hospitals and Ambulance Service. The remuneration varies in each group, but the qualifications and privileges of the staff are the same. The address of the M.A.B. is Victoria Embankment, E.C.—KATIE.

### *THE SICK AND IN NEED.*

**Help for Member of I.S.T.M.**

THE Incorporated Society of Trained Masseuses, 157 Great Portland Street, W., have recently started a Members' Fund, which gives them power to relieve the worst cases of distress amongst their members, and we suggest that you write to the secretary of the society, who will doubtless give you the particulars you require.—MASSEUSE.

**Military Service—Limitation of Liability.**

THE best course for you to adopt when called upon by the recruiting authorities to present yourself for medical examination is to send a written statement of your condition, supported by medical evidence in the form of a certificate from your medical adviser. We have no doubt that due weight will be given to all the facts by the recruiting authorities when considering your case.—A. W.

### *MISCELLANEOUS.*

**Removal of Rust.**

RUST can be readily removed from steel and iron by immersing the instruments in a hot solution of potash and afterwards treating them for a few minutes with dilute hydrochloric acid. A saturated solution of boric acid is useful for this purpose; though not so rapid in action it presents a slight advantage, for when it has dissolved the rust it is slow to attack the metal beneath.—JEAN.

